# Clinical symptoms and chemotherapy completion in elderly patients with newly diagnosed acute leukemia: a retrospective comparison study with a younger cohort

**DOI:** 10.1186/1471-2407-11-224

**Published:** 2011-06-07

**Authors:** Rong Hu, Yong Wu, Xiaoying Jiang, Wenteng Zhang, Le Xu

**Affiliations:** 1 Nursing School of Fujian Medical University. 1 Xueyuan Road, Shangjie Town, Minhou County, Fuzhou, Fujian 350108, P.R. China; 2 Union Hospital Affiliated with Fujian Medical University. 29 Xinquan Road, Fuzhou 350001, Fujian, P.R. China

**Keywords:** Elderly, Acute leukemia, Treatment compliance, Remission

## Abstract

**Background:**

Cancer affects older adults disproportionately. The disease is often difficult to diagnose and treat due to co-morbidities and performance status, and patients tend to discontinue chemotherapy prematurely. There are no systemic studies of the reasons and factors that create a higher withdrawal rate in older acute leukemia patients. This study tried to understand the initial characteristics, blood counts and bone marrow measurements in older acute leukemia patients by comparing them with a younger group to provide information and assistance in early clinical diagnosis, treatment and reasons for treatment withdrawal.

**Methods:**

Using retrospective medical record reviews, we examined clinical characteristics and chemotherapy completion status in the patients of two groups (age ≥ 60, n = 183 and age <60, n = 183) who were diagnosed with acute leukemia for the first time and were hospitalized in Union Hospital Affiliated with Fujian Medical University from 2004 to 2008.

**Results:**

There were no statistical differences in initial presenting symptoms of fatigue (67.2% vs. 57.9%, *P*>0.05) and pallor (53% vs. 59.6%, *P*>0.05) between the two groups, but older patients demonstrated more underlying diseases including lung infections (25.7%, *P *= <0.001), cardiovascular disease (4.4%, *P *= 0.007), and hypertension (20.8%, *P *=< 0.001). The complete remission rate after chemotherapy (1 to 2 courses) was 49.5% in the older group and 66.7% in the younger group (χ^2 ^= 6.202, *P *= 0.013). The percentage of patients age 60 and older who prematurely discontinued chemotherapy (50.3%), mainly due to the influences of traditional Chinese concept of critical illness, financial difficulties, and intolerance to adverse reactions to chemotherapy, was significantly higher than that of younger patients (37.7%) (χ^2 ^= 5.866, *P *= 0.015).

**Conclusions:**

A comprehensive approach to diagnosis, treatment selection, and toxicity management, and implementing strategies to enhance treatment compliance may improve outcomes in older adults with acute leukemia.

## Background

Over the next 20 to 40 years, cancer cases and deaths will double worldwide as populations' age and longevity increases[1]. Cancer disproportionately affects the elderly and as life expectancies increase worldwide, the incidence of malignancy rises[2]. In the United States, death from major tumors by age range from 71-77 years increase dramatically, and the expectation is that other countries will see similar numbers[3]. When cancer occurs in older adults, they tend to have more co-morbidities. Coronary heart disease (CHD), chronic obstructive pulmonary disease (COPD), hypertension, diabetes, non-specific pain, dementia and other diseases of older adults are more common as people age. These co-morbidities often have symptoms similar to that of cancer, leading to potential delays in diagnosis.

The onset of acute leukemia (AL) is often difficult to detect as the disease progresses relatively slowly with few invasive signs and rare early manifestations. Patients often seek medical care because of perceived complications from existing diseases. The diagnosis of disease relies on symptom assessment, laboratory and radiology tests, leading to the potential for missed diagnosis during the early stages of disease in some leukemias. Without prompt diagnosis and treatment, particularly in acute myelogenous leukemia, elderly patients will die of their disease within a short time period of weeks or months[4].

Acute leukemia includes acute lymphocytic leukemia and acute myelogenous leukemia. The remission rate of acute leukemia in elderly patients is low, mortality is high, and survival time is short[5-8]. Induction therapy for acute leukemia is strenuous, and therapy determinations are often made based on age, performance status, and co-morbidities combined with other factors such as cytogenetics[9]. Many older adults are not offered or are not eligible for clinical trials, and most studies are focused on a younger population of patients, rather than elderly patients who are frail. Treatment options for leukemia are more limited in older patients because of co-morbidities[10]. Some studies report an association between age and therapy toxicity in the elderly, but the relationship between age-related side effects and targeted therapies is not clear; and data on the relationship of pharmacokinetics and pharmacodynamics and the impact of age are limited[11]. Even when eligible for treatment, elderly patients may choose palliative care options or prematurely discontinue therapy[10]. Several studies have shown that adherence to therapy was impacted by mood variables and attitude toward cancer[12-14]. Newer targeted therapies may provide new treatment options for older individuals, but additional studies are needed[15].

There is a greater need to understand the implications influencing treatment of AL in the elderly. This study evaluates a series of cases of AL in elderly patients (age ≥ 60) and compares clinical features and characteristics with a younger comparison group (age < 60) in Fujian Province, P.R. China, to determine the rate and causes for patients discontinuing chemotherapy prematurely.

## Methods

The study uses a retrospective comparison design to analyze five years of data captured from archived medical records for patients with acute leukemia in a hospital in Fujian. The study was reviewed and approved by the Ethics Committee of Fujian Medical University. No informed consent was required per the Ethics Committee decision as only patient data were used, patient anonymity was insured by the researchers, and there was no risk to patients.

### Patients

Hospitalized patients treated in the Department of Hematology in Union Hospital Affiliated with Fujian Medical University in Fujian Province from January 2004 to December 2008 with archived medical records in the medical record room were included in the study. Retrospectively reviewed medical record data were used to divide patients into two groups: the elderly group of 183 patients with age ≥ 60 who met inclusion criteria, and the younger group of 183 patients with an age < 60 who met inclusion criteria. Inclusion criteria in the elderly group were previously untreated patients with a cytological examination of bone marrow before treatment began and with results consistent with the diagnostic criteria of acute leukemia (ALL and AML). The diagnostic criteria for acute leukemia were based primarily on clinical characteristics, blood and bone marrow examination by clinical physicians. The confirmed diagnosis was based on bone marrow examination. French-American-British (FAB) classification was used in this study. ALL was classified into L1, L2 and L3 while AML was classified into M0 to M7 subtypes. Case distribution for the elderly group were 28 cases in 2004, 38 cases in 2005, 34 cases in 2006, 47 cases in 2007, and 36 cases in 2008. A total of 183 patients under the age of 60 with acute leukemia were randomly selected as a comparison group using a random number table with an equal number of younger patients as that of elderly patients in each year. No children were included.

### Methods

Archived medical records were reviewed by the researchers and data was recorded to include gender and age, course of disease, initial symptoms, complete blood count with white blood cell differential results at first visit, diagnosis and classification, underlying diseases and complications, chemotherapy situation, chemotherapy regimen ('see Additional file 1: table S1'), treatment course of chemotherapy, whether chemotherapy was stopped during treatment and when, and cause of discontinuing chemotherapy, remission status as defined by Cheson BD, et al. in 2003, [16] and payment method for medical expenses.

### Statistical analysis

Numbers and percentages of categorical variables were analyzed using the chi-square test. When the number of variables in a cell was less than five, Fisher's exact test was used. Multiple logistic regression analysis was used to determine whether the difference between the two groups was still statistically significant after adjusting for select differences in baseline characteristics. Age in the two groups was shown as mean ± standard deviation (SD), and tested using an independent t-test. Statistical significance was considered as two-sided p < 0.05. All data and comparisons were analyzed using SPSS statistical software (version 15.0, SPSS Inc., Chicago, IL).

## Results

### Characteristics at Presentation

A total of 366 medical records were retrospectively reviewed with equal numbers of patient records in the group of patients ≥ 60 years of age (n = 183) and <60 years of age (n = 183). In the younger group, there were 111 males and 72 females with an average age of 34.4 ± 1.0 years old and a range of 15 to 59 years old. In the older group, there were 116 males and 67 females. In the older group, 140 were between 60 to 74 years and 43 were more than 75 years with an average age of 70.1 ± 0.5. The definition of elderly developed by the Asia-Pacific Institute of Studies in October 1980 was used to classify the elderly group as age 60 years or above. Baseline characteristics, co-morbidities, and statistical significance of findings of the elderly group are summarized and compared to the younger group in Table 1. As expected, more patients in the elderly group presented with co-morbidities of lung infection, cardiovascular disease, hypertension and COPD. The younger group presented with a greater incidence of Hepatitis B.

**Table 1:** Patient Characteristics

	Total			Type of Leukemia-ALL^2^			Type of Leukemia-AML^2^		
			
	Younger Group (n = 183)	Elderly Group (n = 183)	p-value^1^	Younger Group (n = 67)	Elderly Group (n = 19)	p-value^1^	Younger Group (n = 116)	Elderly Group (n = 164)	p-value^1^
Age (years)	34.4 ± 13.1	70.1 ± 6.4	<0.001*	30.2 ± 13.3	69.3 ± 5.5	<0.001*	36.8 ± 12.4	70.2 ± 6.5	<0.001*
Male gender	111 (60.7)	116 (63.4)	0.59	46 (68.7)	10 (52.6)	0.196	65 (56.0)	106 (64.6)	0.146
Married	131 (71.6)	183 (100.0)	<0.001*	34(50.7)	19 (100)	<0.001*	97 (83.6)	164 (100)	<0.001*
Smoking	14 (7.7)	34 (18.6)	0.002*	3 (4.5)	3 (15.8)	0.122	11 (9.6)	31 (18.9)	0.032*
Alcohol use**	4 (2.2)	12 (6.6)	0.041*	1 (1.5)	0 (0)	1.000	3 (2.6)	12 (7.3)	1.000
History of carcinoma***	4 (2.2)	4 (2.2)	1.000	0 (0)	1 (5.3)	0.221	4 (3.4)	3 (1.8)	0.454
Lung infection	18 (9.8)	47 (25.7)	<0.001*	3 (4.5)	3 (15.8)	0.119	15 (12.9)	44 (26.8)	0.005*
Cardiovascular disease	0 (0.0)	8 (4.4)	0.007*	0 (0)	0 (0)	-	0 (0)	8 (4.9)	0.023*
Hypertension	3 (1.6)	38 (20.8)	<0.001*	2 (3.0)	5 (26.3)	0.005*	1 (0.9)	33 (20.1)	<0.001*
Diabetes	4 (2.2)	14 (7.7)	0.016*	0 (0)	2 (10.5)	0.047*	4 (3.4)	12 (7.3)	0.200
COPD	0 (0.0)	9 (4.9)	0.004*	0 (0)	0 (0)	-	0 (0)	9 (5.5)	0.012*
Hepatitis B	14 (7.7)	8 (4.4)	0.187	9 (13.4)	3 (15.8)	0.723	5 (4.3)	5 (3.0)	0.575

Data were represented as n (%) except age was as mean ± SD

SD = standard deviation; COPD = chronic obstructive pulmonary disease

^1 ^Chi-square test or Fishers' exact test was used for categorical data and independent t-test was used for age.

^2 ^The types were based on French-American-British classification.

*Indicates significant differences between two groups, p < 0.05.

**Consuming 5 or more drinks per day.

***History of other malignant tumors occurring before the diagnosis of acute leukemia.

### Classification of Disease and Symptoms

Comparisons of initial manifestations between the elderly patient group and the younger patient group are shown in Table 2 and include the types of acute leukemia in French-American-British classification, initial symptoms, hematology, and degree of cellularity of bone marrow. Compared with younger patients, more elderly patients had acute myeloid leukemia [AML, 89.6% (164/183) vs. 63.4% (116/183)]. Fewer elderly patients had extremely active bone marrow cellularity (33.3% vs. 49.2%). The initial manifestations of disease in elderly patients were fatigue (67.2%) and pallor (53.0%) as compared to pallor (59.6%) and fatigue (57.9%) in the younger group.

**Table 2:** Type of Acute Leukemia, Initial Symptoms, Hematology and Degree of Bone Marrow Cellularity

	Total			Type of Acute Leukemia-ALL			Type of Acute Leukemia-AML		
			
Initial symptom	Younger Group (n = 183)	Elderly Group (n = 183)	p-value^1^	Younger Group (n = 67)	Elderly Group (n = 19)	p-value^1^	Younger Group (n = 116)	Elderly Group (n = 164)	p-value^1^
Hypodynamia	106 (57.9)	123 (67.2)	0.066	39 (58.2)	11 (57.9)	0.980	67 (57.8)	112 (68.3)	0.071
Pale face	109 (59.6)	97 (53.0)	0.206	39 (58.2)	6 (31.6)	0.040*	70 (60.3)	91 (55.5)	0.418
Fever	78 (42.6)	61 (33.3)	0.067	31 (46.3)	6 (31.6)	0.254	47 (40.5)	55 (33.5)	0.232
Hemorrhagic dermatologic mucosa	25 (13.7)	25 (13.7)	1.000	7 (10.4)	2 (10.5)	1.000	18 (15.5)	23 (14.0)	0.728
Dizziness	29 (15.8)	20 (10.9)	0.167	12 (17.9)	3 (15.8)	1.000	17 (14.7)	17 (10.4)	0.279
WBC infiltration**	17 (9.3)	24 (13.1)	0.246	9 (13.4)	4 (21.1)	0.471	8 (6.9)	20 (12.2)	0.145
Lower extremity edema	0(0)	5(2.73)	0.024	0 (0)	2 (10.5)	0.047*	0 (0)	3 (1.8)	0.269
Myalgias	5(2.73)	5(2.73)	1.000	0 (0)	0 (0)	-	5 (4.3)	5 (3.0)	0.575
White cell count (10^9^/L)			0.139			0.057			0.012*
<4	56 (30.6)	69 (37.7)		28 (41.8)	3 (15.8)		28 (24.1)	66 (40.2)	
4 - 10	29 (15.8)	18 (9.8)		10 (14.9)	2 (10.5)		19 (16.4)	16 (9.8)	
>10	98 (53.6)	96 (52.5)		29 (43.3)	14 (73.7)		69 (59.5)	82 (50.0)	
Hemoglobin (g/L)^2^			0.549			0.078			0.336
Abnormal	168 (91.8)	171 (93.4)		60 (89.6)	14 (73.7)		108 (93.1)	157 (95.7)	
Normal	15 (8.2)	12 (6.6)		7 (10.4)	5 (26.3)		8 (6.9)	7 (4.3)	
Platelets (10^9^/L)			0.018*			0.273			0.127
≦ 10	59 (32.2)	39 (21.3)		25 (37.3)	4 (21.1)		34 (29.3)	35 (21.3)	
>10	124 (67.8)	144 (78.7)		42 (62.7)	15 (78.9)		82 (70.7)	129 (78.7)	
Bone marrow cellularity			0.014*			0.400			0.002*
Severely hypercellular	90 (49.2)	61 (33.3)		28 (41.8)	11 (57.9)		62 (53.4)	50 (30.5)	
Moderately hypercellular	38 (20.8)	58 (31.7)		13 (19.4)	1 (5.3)		25 (21.6)	57 (34.8)	
Normocellular	31 (13.1)	39 (21.3)		15 (22.4)	5 (26.3)		16 (13.8)	34 (20.7)	
Hypocellular	24 (13.1)	25 (13.7)		11 (16.4)	2 (10.5)		13 (11.2)	23 (14.0)	

^1 ^Chi-square test was used for categorical data.

^2 ^Abnormal was defined as hemoglobin 110 g/L for female and hemoglobin 120 g/L for male.

*Indicates significant differences between two groups, p < 0.05.

**White blood cell infiltration

### Remission Status and Complications

Table 3 shows discontinuation of chemotherapy, remission status and complications caused by chemotherapy. In the elderly group, 92 patients (50.3%) withdrew from chemotherapy (45 patients did not receive chemotherapy after diagnosis; 39 patients withdrew after the first chemotherapy cycle; 8 patients withdrew after the second chemotherapy cycle). In the younger group, a total of 69 patients (37.3%) withdrew from chemotherapy (29 patients did not receive chemotherapy after diagnosis; 32 patients withdrew after the first chemotherapy cycle; 8 patients withdrew after the second chemotherapy cycle). There was significant difference between the two groups in discontinuing chemotherapy (*P *= 0.015). Younger patients completing therapy had a significantly higher rate of complete remission (66.7%) than elderly patients (49.5%). Interestingly, the difference in remission status was only significant in patients with AML if the acute leukemia classification was considered (Table 4). Remission status stratified by type of leukemia and completion or non-completion of therapy is shown in Table 4. As for complications from chemotherapy, a higher percentage of elderly patients suffered from cardiotoxicity (7.1% vs. 0.5%) and lung infection (49.2% vs. 29.0%) when compared to younger patients. Multiple logistic regression analysis was used to adjust for smoking, drinking, lung infection, hypertension, diabetes and other factors.

**Table 3:** Remission Status and Complications Caused by Chemotherapy

	**Total**				**Type of Leukemia-ALL**				**Type of Leukemia-AML**			
			
**Variables**	**Younger** **Group** **(n = 183)**	**Elderly** **Group** **(n = 183)**	**p-value^1^**	**Adjusted** **P^3^**	**Younger** **Group** **(n = 67)**	**Elderly** **Group** **(n = 19)**	**p-value^1^**	**Adjusted P^4^**	**Younger** **Group** **(n = 116)**	**Elderly** **Group** **(n = 164)**	**p-value^1^**	**Adjusted P^4^**
		
Chemotherapy dropout	69 (37.7)	92 (50.3)	0.015*	-	20 (29.9)	9 (47.4)	0.154	-	49 (42.2)	83 (50.6)	0.167	-
Remission status^2^			0.013*	0.008*			0.415	0.422			0.018	0.010*
Partial	38 (33.3)	46 (50.5)			17 (36.2)	5 (50.0)			21 (31.3)	41 (50.6)		
Complete	76 (66.7)	45 (49.5)			30 (63.8)	5 (50.0)			46 (68.7)	40 (49.4)		
Complication caused by chemotherapy												
Mouth ulcer	31 (16.9)	23 (12.6)	0.238	0.728	13 (19.4)	3 (15.8)	1.000	0.899	18 (15.5)	20 (12.2)	0.424	0.642
Gastrointestinal disorder	34 (18.6)	37 (20.2)	0.692	0.170	18 (26.9)	5 (26.3)	0.962	0.629	16 (13.8)	32 (19.5)	0.211	0.127
Cardiotoxicity	1 (0.5)	13 (7.1)	0.001*	0.047*	0 (0)	1 (5.3)	0.221	-	1 (0.9)	12 (7.3)	0.017	0.068
Liver damage	26 (14.2)	29 (15.8)	0.661	0.607	9 (13.4)	5 (26.3)	0.179	0.228	17 (14.7)	24 (14.6)	0.996	0.987
Lung infection	53 (29.0)	90 (49.2)	<0.001*	0.024*	13 (19.4)	9 (47.4)	0.014	0.025	40 (34.5)	81 (49.4)	0.013	0.138
Angina	89 (48.6)	74 (40.4)	0.115	0.698	33 (49.3)	8 (42.1)	0.582	0.827	56 (48.3)	66 (40.2)	0.182	0.669
Intestinal infection	38 (20.8)	36 (19.7)	0.795	0.951	17 (25.4)	4 (21.1)	1.000	0.538	21 (18.1)	32 (19.5)	0.767	0.871
Fungal infection	36 (19.7)	51 (27.9)	0.065	0.285	10 (14.9)	6 (31.6)	0.100	0.051	26 (22.4)	45 (27.4)	0.341	0.755
Skin hemorrhage	48 (26.2)	29 (15.8)	0.013*	0.019*	14 (20.9)	4 (21.1)	1.000	0.689	34 (29.3)	25 (15.2)	0.004	0.015*

^1 ^Chi-square test was used.

^2 ^The remission status was for those patients who finished the whole chemotherapy.

^3 ^Multiple logistic regression analysis that adjusted for smoking, alcohol use, lung infection, hypertension, diabetes, type of leukemia, platelet and bone marrow cellularity

^4 ^Multiple logistic regression analysis that adjusted for smoking, alcohol use, lung infection, hypertension, diabetes, platelet and bone marrow cellularity

* Indicates significant difference between two groups, p < 0.05.

**Table 4:** Remission Status Stratified by FrenchAmerican-British Classification

	ALL				AML			
		
	Elderly Group (n = 19) N (%)	Younger Group (n = 57) N (%)	p-value^1^	adjusted p^2^	Elderly Group (n = 164) N (%)	Younger Group (n = 116) N (%)	p-value^1^	adjusted p^2^
Remission			0.274	0.422			0.022*	0.010*
status								
Partial	5 (26.3)	17 (25.4)			41 (25.0)	21 (18.1)		
Complete	5 (26.3)	30 (44.8)			40 (24.4)	46 (39.7)		

ALL = acute lymphoblastic leukemia; AML = acute myeloid leukemia

^1 ^Chi-square test was used.

^2 ^Multiple logistic regression analysis that adjusted for smoking, alcohol use, lung infection, hypertension, diabetes, type of leukemia, platelet and bone marrow cellularity

* Indicates significant differences between two groups, p < 0.05.

### Causes of Discontinuing Chemotherapy

Approximately one half of elderly patients stopped chemotherapy before completing therapy (50.3%) while there were 37.7% younger patients discontinued chemotherapy. The main reasons that led to discontinuing chemotherapy in elderly group were increasing severity of leukemia symptoms (42.4%), economic difficulty (37.0%), and the patient's inability to tolerate adverse effects (12.0%) (Table 5). The main reason for younger patients to discontinue chemotherapy was economic difficulty (66.7%), next reason was increasing severity of leukemia symptoms (21.7%).

**Table 5:** Causes of Chemotherapy Discontinuations in Elderly and Younger Group Patients

Causes	Young (n = 69)*	Elderly (n = 92)*	P value**
Severe clinical AL symptoms	15 (21.7)	39 (42.4)	0.06
Economic difficulty	46 (66.7)	34 (37.0)	<0.001
Could not tolerate adverse effects	2 (2.9)	11 (12.0)	0.037
Unknown	6 (8.7)	8 (8.6)	0.896

*: The data were presented as number (%)

**: The χ^2 ^test was used for the statistical analysis.

## Discussion

Acute leukemia in elderly patients is a severe, high-risk hematological disease whose incidence is increasing with the age of the population[17]. Survey data in the United States in 2006 (Surveillance, Epidemiology and End Results, SEER) showed that the incidence rate of acute myeloid leukemia (AML) was 16.9/10 million in the population 65 years and over[18]. Survival rates have not improved for older patients with acute leukemia, in part because they may not be offered aggressive therapies and clinical trials, or they may choose to forego chemotherapy or choose to stop therapy before completion[6,9]. This study shows that older patients (≥ 60) present with more co-morbidities than younger patients, have a poorer remission status and more complications of chemotherapy, and are more likely to stop chemotherapy before completing prescribed cycles due to severity of disease and economic factors. As the age of acute leukemia patients increases, adherence to chemotherapy regiment decreases[13]. Our findings suggest that a careful history and physical examination at presentation, treatment selection based on multiple factors rather than age, aggressive management of complications of therapy and co-morbidities, and sufficient support resources to remain in therapy are needed to improve outcomes in older leukemia patients.

The performance status of a patient may determine the type of induction therapy offered. If a patient is perceived as too frail because of co-morbid conditions or age, supportive care without chance for remission may be the only option, but investigating less toxic regimens or clinical trials may offer acceptable alternatives[4]. The initial symptoms of the two groups included fatigue, pallor, fever, skin and mucous membrane bleeding, lower extremity edema, and dizziness. There were no significant differences between the two groups with the exception of lower extremity edema. In elderly patients, the co-morbidities of hypertension, coronary heart disease, COPD, diabetes, and liver disease may have similar presenting symptoms to that of acute leukemia. A diagnosis of acute leukemia may be missed in clinical practice based on the similarity of presentations without extensive laboratory and other testing. While a literature search revealed no specific studies related to co-morbidities masking the onset of leukemia, one case report described a patient with persistent cervical lymphadenopathy with an initial diagnosis of toxoplasmosis that masked non-Hodgkin's lymphoma[19]. Disease onset is often less evident in elderly patients who describe multiple non-specific symptoms, and patients and families may not pay attention to described symptoms leading to a delay in diagnosis. A delay in seeking treatment may lead to fewer options for the patient.

Complete blood count monitoring in elderly patients is important in clinical practice for early diagnosis and treatment of a variety of diseases, including leukemia. Pancytopenia in peripheral blood was common in elderly patients with newly diagnosed acute leukemia. Varying degrees of anemia occurred in 93.44% of elderly patients and 16.94% of elderly patients experienced severe anemia, which were not significantly different than that of younger patients. In elderly patients, the platelet count was lower than normal, with 21.31% of patients with platelets lower than 10 × 10^9 ^/L. The percentage of younger patients whose platelet count was less than 10 × 10^9 ^/L was 32.2%, which was significantly different from that of elderly patients. The degree of bone marrow cellularity in elderly patients was significantly reduced compared with that of younger patients (33.3% vs. 49.2%) (Table 2).

This study found that AML accounted for 89.62% of the leukemias in the elderly patient group, with M5 (acute monocytic leukemia) as the most common, accounting for 48.63% of cases. ALL (acute lymphoblastic leukemia) represented the remaining 10.38% in the elderly patient group. In the younger group, the incidence of ALL was higher (36.61%); with AML accounting for 63.4% of cases, of which M5 accounted for 23.50%. Our findings are similar to literature findings that more than half of diagnosed AML patients are over 60 years of age[17].

The complete remission rate was 49.45% for all participants in the elderly patient group after one to two courses of regular chemotherapy which was significantly lower than that of the younger patient group (66.67%)(Table 3). This was consistent with most reports in the literature[9]. Reasons may include that elderly patients have less immunity, may have drug resistance due to physiologic changes associated with aging, often exhibit more underlying diseases and cardio-pulmonary complications, their hematopoietic function recovers slowly, and combination chemotherapy has significant side effects. Some elderly patients only agreed to simple examinations at the hospital, and refused bone marrow biopsy, resulting in delayed treatment. When symptoms progressed to high fever, bleeding, severe anemia, weight loss, and failure to thrive, the optimal timing for treatment was missed. Carefully explaining the reason for diagnostic testing and examinations may mitigate some treatment refusals when the elderly patient presents with symptoms. Physiological age should be considered as well as chronological age in treatment choices[11]. Future studies of newer agents with less severe side effects and studies of clinicopharmacologic implications of drug therapy in older patients are indicated.

The results of this study also showed that the percentage of elderly patients with acute leukemia who discontinued chemotherapy was 50.27%, which was significantly higher than that of the younger group (37.3%). Family members often worry that patients may not tolerate chemotherapy. Traditional Chinese beliefs in families lead to the hope that elderly patients can die at home, leading to a request for discharge from the hospital rather than aggressive treatment in some cases. In a study by Alam et al.[13], it was reported that Canadian patients discontinued chemotherapy prematurely in greater numbers than American patients. Considering our results and that of other studies, the question of whether cultural and national differences influence therapy discontinuation should be investigated.

Financial difficulties were the second most common reason (36.96%) that elderly patients discontinued chemotherapy prematurely, while 66.7% of younger patients discontinued chemotherapy for financial constraints. Treatment of acute leukemia often leads to high medical cost. Currently many families cannot afford high medical expenses as the National Health Care system and the Social Welfare system have not been fully established in China. In one study, patients from lower socio-economic classes had poorer survival perhaps due to class bias, greater co-morbidities, or ability to pay[20]. Psychological distress in cancer patients may impact treatment decisions and additional studies are warranted[21]. Patients and their families may choose to give up treatment if costs cannot be managed. Identifying social support measures may mitigate financial difficulties as a reason for stopping chemotherapy.

Studies in the literature state that proactive identification of patients at high risk of side effects is important and aggressive management is crucial[2]. A discussion of the multiple options for treatment of acute leukemia with the patient and family is also important to select a regimen that delivers the best possible outcome with the least toxic side effects[9]. Of the 50.27% of older newly diagnosed AL patients who discontinued therapy in this study, 11.96% of elderly patients discontinued chemotherapy during the first or second course because they could not tolerate the side effects of chemotherapy. In addition to decreased normal physiological functions, prognosis of acute leukemia in elderly patients is affected by multiple poor prognostic factors. One third of older patients experience combined vital organ diseases including heart, brain, and kidney diseases, and a lower tolerance and slower clearance of cytotoxic drugs; so toxic side effects may be increased accordingly[2]. Patients exhibit different tolerances to chemotherapy based on individual factors, and patients who are sensitive to chemotherapy may still refuse treatment because of the seriousness of side effects induced by chemotherapy.

Univariate analysis in this study also showed that adherence to chemotherapy was poorer in older patients. The percentage of elderly patients who discontinued chemotherapy (50.27%) was significantly higher than in younger patients (37.3%) (Table 3). In some cases, family members could not deal with patients' suffering, and requested discharge from the hospital as soon as the disease condition was improved slightly. Patients either remained at home after discharge or were admitted to their local hospital. Older patients sometimes felt their lives were less valuable. They requested discharge and discontinuation oftreatment because there was no improvement in their illness even when they spent much of their savings.

Limitations of the study include the retrospective nature of medical record reviews, limited demographic information, lack of stratification by treatment regimen in group comparisons, the fact that the two groups were not matched other than by general diagnosis, and there were no defined criteria to measure and compare toxicity. Efforts should be made to determine if cultural factors play a more significant role than evident in our study. Data was not captured that indicated whether the patient or family made the decision to discontinue chemotherapy. Future studies should incorporate a prospective design, define additional data points for study, and consider incorporating additional information requests about non-clinical reasons for treatment decisions.

## Conclusion

Acute monocytic leukemia is the most common acute leukemia affecting elderly patients and cases of acute leukemia in the older population are rising. In our study we found that disease onset is less evident, and initial manifestations include fatigue and pallor, symptoms that can indicate multiple disorders. In some elderly patients, the disease is characterized by leukemia with low proliferative activity, pancytopenia, and bone marrow hypoplasia, which may easily cause misdiagnosis or missed diagnosis. A comprehensive history and physical, including appropriate laboratory and other diagnostic tests are indicated. Performance status and co-morbidities should be considered in selecting an appropriate treatment regimen with attention to potential toxicity for the older patient. Compliance to chemotherapy regimens is poor in elderly patients and the percentage of patients discontinuing chemotherapy is high. Thoughtful treatment regimen selection to balance potential toxicity as well as cost, prompt attention to side effects and toxicities during treatment, and support for non-clinical issues such as cultural beliefs, quality of life and cost of therapy are important in the care of the elderly patient with acute leukemia. Additional studies are needed to further explore factors affecting elderly patients' decisions to initiate or stop acute leukemia treatment.

## Competing interests

The authors declare that they have no competing interests.

## Authors' contributions

RH initiated the study concept and design and performed the data collection and analysis; YW did the data collection and analysis and technical support; XJ performed study design and supervision; WZ attended the data collection; LX attended the study supervision. All authors read and approved the final manuscript.

## Pre-publication history

The pre-publication history for this paper can be accessed here:

http://www.biomedcentral.com/1471-2407/11/224/prepub

## Supplementary Material

Additional file 1 **table S1: Schedules and chemotherapy agents for treatment of AML and ALL patients**. Additional file 1 contains a table about Schedules and chemotherapy agents for treatment of AML and ALL patients Click here for file
