# Supplementary material for: Clinical symptoms and chemotherapy completion in elderly patients with newly diagnosed acute leukemia: a retrospective comparison study with a younger cohort
Source: BMC Cancer. 2011 Jun 7;11:224. doi: 10.1186/1471-2407-11-224 (PMC3130702; doi:10.1186/1471-2407-11-224)
Supplement: Additional file 1 — table S1: Schedules and chemotherapy agents for treatment of AML and ALL patients. Additional file 1 contains a table about Schedules and chemotherapy agents for treatment of AML and ALL patients [file 1471-2407-11-224-S1.PDF]

Table S1: Schedules and chemotherapy agents for treatment of AML and ALL patients.

Schedule	Drugs	Course
ALL		
VDLP	vincristine 1.5 mg/m^2 Ⅳ at 1，8，15，21 day	28 days
	daunorubicin (DNR) 30-40mg/m^2 Ⅳ on day 1 to 3 and day 15 to 17	
	L-asparaginase 6000u/m^2 Ⅳ on day 19 to 28	
	prednisone 40-60mg/m^2 orally on days 1 to 28	
HD-MTX	Methotrexate (MTX）1-1.5g/m^2 IV at 24h to protect with calcium folinate within 12h after the drugs. 6－9mg/m^2 Q 6h for 8 times	
AML		
DA	daunorubicin (DNR) 30-40mg/m^2 Ⅳ on day 1,2 and 3	7 days
	Cytarabine (Ara-C) 100-150 mg/m^2 Ⅳ on day 1 to 7	
HA	Harringtonine 3-4mg/m^2 Ⅳ on day 1 to 7	7 days
	Cytarabine (Ara-C) 100-150mg/m^2 Ⅳ on day 1 to 7	
DAE	daunorubicin 30-60mg/m^2 Ⅳ on day 1 to 3	7 days
	Cytarabine (Ara-C) 100-150 mg/m^2 Ⅳ on day 1 to 7	
	VP16 75 mg/m^2 Ⅳ on day 5 to 7	
MA	Mitoxantrone 5 mg/m^2 Ⅳ on day 1 to 3	7 days
	Cytarabine (Ara-C) 100-150 mg/m^2 Ⅳ on day 1 to 7	
D H－A	daunorubicin (DNR) 30-40 mg/m^2 Ⅳ on day 1 to 3	7 days
	Cytarabine (Ara-C) 1.0g/m^2 Ⅳ,Q12h day 1 to 3	
H H－A	Harringtonine 3-4mg/m^2 Ⅳ on day 1 to 7	7 days
	Cytarabine (Ara-C) 1.0g/m^2 Ⅳ,Q12h day 1 to 3	
